# Maternal Mental Health and Children’s Problem Behaviours: A Bi-directional Relationship?

**DOI:** 10.1007/s10802-023-01086-5

**Published:** 2023-07-04

**Authors:** Emily Lowthian, Stuart Bedston, Sara Madeleine Kristensen, Ashley Akbari, Richard Fry, Katy Huxley, Rhodri Johnson, Hyun Sue Kim, Rhiannon K. Owen, Chris Taylor, Lucy Griffiths

**Affiliations:** 1 Population Data Science, Swansea University Medical School, Swansea University, Singleton Park, Swansea, SA2 8PP Wales; 2 School of Social Sciences, Population Data Science, Swansea University Medical School, Swansea University, Wales, UK; 3 Department of Health Promotion and Development, University of Bergen, Bergen, 5020 Norway; 4 Department of Education and Childhood Studies, School of Social Sciences, Swansea University, Swansea, SA2 8PP Wales; 5 Virginia Tech Carilion School of Medicine, 2 Riverside Circle, Roanoke, VA 24016 United States

**Keywords:** Maternal mental health, Child development, Structural equation modelling, Millennium Cohort Study, Bayesian analysis

## Abstract

Transactional theory and the coercive family process model have illustrated how the parent-child relationship is reciprocal. Emerging research using advanced statistical methods has examined these theories, but further investigations are necessary. In this study, we utilised linked health data on maternal mental health disorders and explored their relationship with child problem behaviours via the Strengths and Difficulties Questionnaire for over 13 years. We accessed data from the Millennium Cohort Study, linked to anonymised individual-level population-scale health and administrative data within the Secure Anonymised Information Linkage (SAIL) Databank. We used Bayesian Structural Equation Modelling, specifically Random-Intercept Cross-Lagged Panel Models, to analyse the relationships between mothers and their children. We then explored these models with the addition of time-invariant covariates. We found that a mother’s mental health was strongly associated over time, as were children’s problem behaviours. We found mixed evidence for bi-directional relationships, with only emotional problems showing bi-directional associations in mid to late childhood. Only child-to-mother pathways were identified for the overall problem behaviour score and peer problems; no associations were found for conduct problems or hyperactivity. All models had strong between-effects and clear socioeconomic and sex differences. We encourage the use of whole family-based support for mental health and problem behaviours, and recommend that socioeconomic, sex and wider differences should be considered as factors in tailoring family-based interventions and support.

**Supplementary Information:**

The online version contains supplementary material available at 10.1007/s10802-023-01086-5.

## Introduction

Maternal mental health represents one of the earliest influences on a child’s development, and research shows that maternal mental illness may negatively affect child development, behaviour, cognitive, and socio-emotional development (Kingston & Tough, 2014) even after adjustment for key covariates such as socioeconomic status (Mensah & Kiernan, 2010). While a considerable body of research has examined how parental mental illness can affect children’s mental health (Goodman et al., 2011; Kingston & Tough, 2014; Reupert & Maybery, 2016; Yamamoto & Keogh, 2018) and access to services (Acri et al., 2017; Fledderjohann et al., 2021; Haine-Schlagel et al., 2018; Pfefferle & Spitznagel, 2009), fewer studies assess how child problem behaviours impact parental mental health (Goodman et al., 2011; Xerxa et al., 2021). There is a need to consider the complex interplay of interactions between parent and child, labelled ‘bi-directional associations’ by Bell (1968, 1971) and Sameroff (1975). In this paper, we draw on transactional theory and findings from recent, methodologically advanced studies to inform our investigation.

### Transactional Theory

Interactions between the parent and child have been largely influenced by Bell (1968), who provided a catalyst for research exploring the two-way exchange of behaviours from parent to child, and vice-versa (Pettit & Arsiwalla, 2008). Bell (1971) explained that seeing children as passive recipients of their interactions with others is a limited perspective as it fits a one-sided model of parental determination of behaviour (Sameroff, 1975). Rather than viewing children as a static environment, there is a recognition that children are in a “perpetual state of active reorganization” (Sameroff, 1975, p. 8), and their development is shaped by the environment *and* their interaction with an experience. In a *transactional* conceptualisation of risk, it is recognised that proximal and distal factors occur in a dynamic interplay, whereby the child is influenced by the environment *yet* simultaneously responds to the environment and so forth (Cicchetti & Toth, 1997). As life progresses, they are driven by new complexities at both the individual and experience levels, and these require new adaptations (Sameroff, 2009).

Within parent-child relationships, the labelling of a child by a parent as ‘difficult’ may result in persistent labelling regardless of actual behaviour. In turn, a child may accept this labelling and increase difficult behaviour (Sameroff, 1975). This is often referred to as the Coercive Process Model (Patterson, 1982, 2002a). Belsky (1984) further developed the Process Model giving greater recognition to how child characteristics influenced parenting. For example, in Field et al. (1988) infants (aged 3–6 months) of mothers with depressive symptoms showed greater ‘depressed’ behaviour even when interacting with mothers without depressive symptoms, and the ‘non-depressed’ group of mothers began to exhibit depressive manners in interactions with infants.

The understanding of bi-directional relationships has advanced into other fields, such as parental mental health or psychopathology and child problems (e.g., Gross et al., 2008; Xerxa et al., 2021), recognising the mutual influence between a child and their environment as a continuous dynamic interacting with the social context (similar to e.g., Pettit & Arsiwalla 2008). Building on Gross et al. (2008) maternal depression compromises parenting (Galbally & Lewis, 2017; Vreeland et al., 2019) which is associated with increased child problems (Middleton et al., 2009; Riany et al., 2022; Sweeney & MacBeth, 2016). Subsequently, parents experience a decrease in their parenting competency, which may prolong or intensify the feelings of depression (Cutrona & Troutman, 1986; Nelson et al., 2003). Alongside the complex relationship between parent and child exists the wider environment, including structural determinants such as socioeconomic status. For example, Sameroff (2000) explains how maternal mental health problems can often sit within the contexts of poverty, lack of social support, stressful life events and low cultural capital; the theory that advantaged families (economically and socially) have greater access to knowledge and systems which promote positive child outcomes (Bourdieu, 1985). Consequently, the individual, family, and wider environmental factors combine to influence child development (Bronfenbrenner, 1979).

### Recent Findings and Advanced Methodologies

In recent years, more advanced methodological techniques have been used to further quantify and test bi-directional associations between parental and child problems (Cortés-García et al., 2019; Hickey et al., 2020; Speyer et al., 2022; Xerxa et al., 2021). In terms of parental mental health and child development specifically, Xerxa et al. (2021) found bi-directional associations between maternal and paternal psychopathology and child behaviour problems using parent-reported data from the Netherlands (internalising and externalising symptoms). Associations were observed where the parent reported on both their own psychopathology symptoms and the child’s behaviour (termed as within-rater). But, there were either weak or no associations when examining maternal psychopathology and the paternal reports of child behaviour problems, nor between paternal psychopathology and maternal reports of child behaviour problems (termed as shared-rater). This illuminates the bias with parental reports of child behaviour. Speyer et al. (2022), using the Millennium Cohort Study (MCS) in the UK, examined parental distress and child behaviour problems (internalising and externalising symptoms) and found that maternal distress was associated with child internalising symptoms, while paternal distress was associated with increased problems for boys and decreased problems for girls. Bi-directional associations showed that internalising symptoms in boys and externalising symptoms in girls increased maternal distress; conversely, internalising symptoms in girls and externalising symptoms in boys increased paternal distress. There is, then, mixed evidence for understanding bi-directional associations.

Emerging evidence also suggests that providing care for a child who has hyperactivity problems provides additional demands and challenges (Johnston & Mash, 2001). While evidence suggests that parental factors are associated with the development of ADHD (Attention Deficit Hyperactivity Disorder) or other areas of hyperactivity (Wüstner et al., 2019), less is known about the reverse. A longitudinal study by Breaux and Harvey (2019) found that child ADHD symptoms significantly predicted maternal depression symptoms and vice-versa; for fathers, only child effects were found with ADHD symptoms predicting paternal depression across the pre-school years. However, this study used cross-lagged panel models without a random intercept to estimate between-effects which has been criticised (Hamaker et al., 2015).

Other areas of child problem behaviours include interaction with their peers. Evidence suggests that maternal depression is associated with greater child peer problems (Waerden et al., 2015). A key mechanism in this relationship is parenting, wherein Wang et al. (2021) found no authoritative parenting practices (balance of warmth and control) in a group of Chinese parents with major depressive disorder, but identified authoritarian and permissive styles, which are considered less desirable; note however, that parenting is sensitive to cultural contexts (Stewart & Bond, 2002). Moreover, Yamagata et al. (2013) found bi-directional associations between parenting and peer problems independent of genetic and family covariates in a path analysis of 259 pairs of twins in early childhood. As parenting strain can be related to parental mental health (Borre & Kliewer, 2014), our study will additionally explore if children’s peer problems are related to maternal depression and then vice-versa.

In addition to the limited and mixed picture on bi-directional associations, further areas require investigation. First, despite some studies using clinical measures, the research base often uses self-reports of parental mental health to capture constructs of well-being (Mensah & Kiernan, 2010; Speyer et al., 2022; Xerxa et al., 2021). The use of these risk social desirability bias (Hunt et al., 2003; Sigmon et al., 2005) or responses depicting short-term mood, and in some data, self-reports had a lower prevalence of mental health problems than administrative records (O’Donnell et al., 2016). This is particularly significant when cohort studies capture the parent and child report on the same day – leading to problems with measurement bias (Najman et al., 2000; Ringoot et al., 2015). In addition, child sex has traditionally been a strong focus in bi-directional research, although factors such as socioeconomic status are also strongly associated with mental health in both adults (Finegan et al., 2018; Marmot & Bell, 2012) and children (Reiss, 2013) but often does not feature in much research. Therefore, studies should consider alternative measures of parental mental health and include key structural covariates in modelling to address these existing gaps.

### The Present Study

This study examined the bi-directional associations between primary care maternal mental health records and parent-reported child problem behaviours. We adjusted for child sex and socioeconomic status to explore if bi-directional associations are sensitive to key individual and structural determinants. We also investigated the full range of child problems to explore associations in emotional and conduct problems, but also hyperactivity and peer problems, which are less developed in the field. We combined data from the Millennium Cohort Study (MCS) with anonymised individual-level population-scale health administrative data from the Secure Anonymised Information Linkage (SAIL) Databank in Wales.

Based on the theoretical assumptions in transactional theory (Bell, 1968, 1971; Belsky, 1984; Sameroff, 1975, 2000) and the coercive family process model (Patterson, 1982) and previous research in the field (e.g., Cortés-García et al., 2019; Hickey et al., 2020; Speyer et al., 2022; Xerxa et al., 2021), we hypothesised the following:

1. Maternal mental health problems and children’s emotional, behavioural, hyperactivity, and peer problems are positively and concurrently related at a between- and within-person level.
2. There is a positive carry-over stability effect in children’s emotional, behavioural, hyperactivity, and peer problems (i.e., deviation from one’s own norm is associated with a similar deviation at subsequent time points).
3. Maternal mental health problems are positively associated with children’s subsequent emotional, behavioural, hyperactivity, and peer problems.
4. Children’s emotional, behavioural, hyperactivity, and peer problems are positively related to later maternal mental health problems.

### Data and Methods

**Fig. 1:**
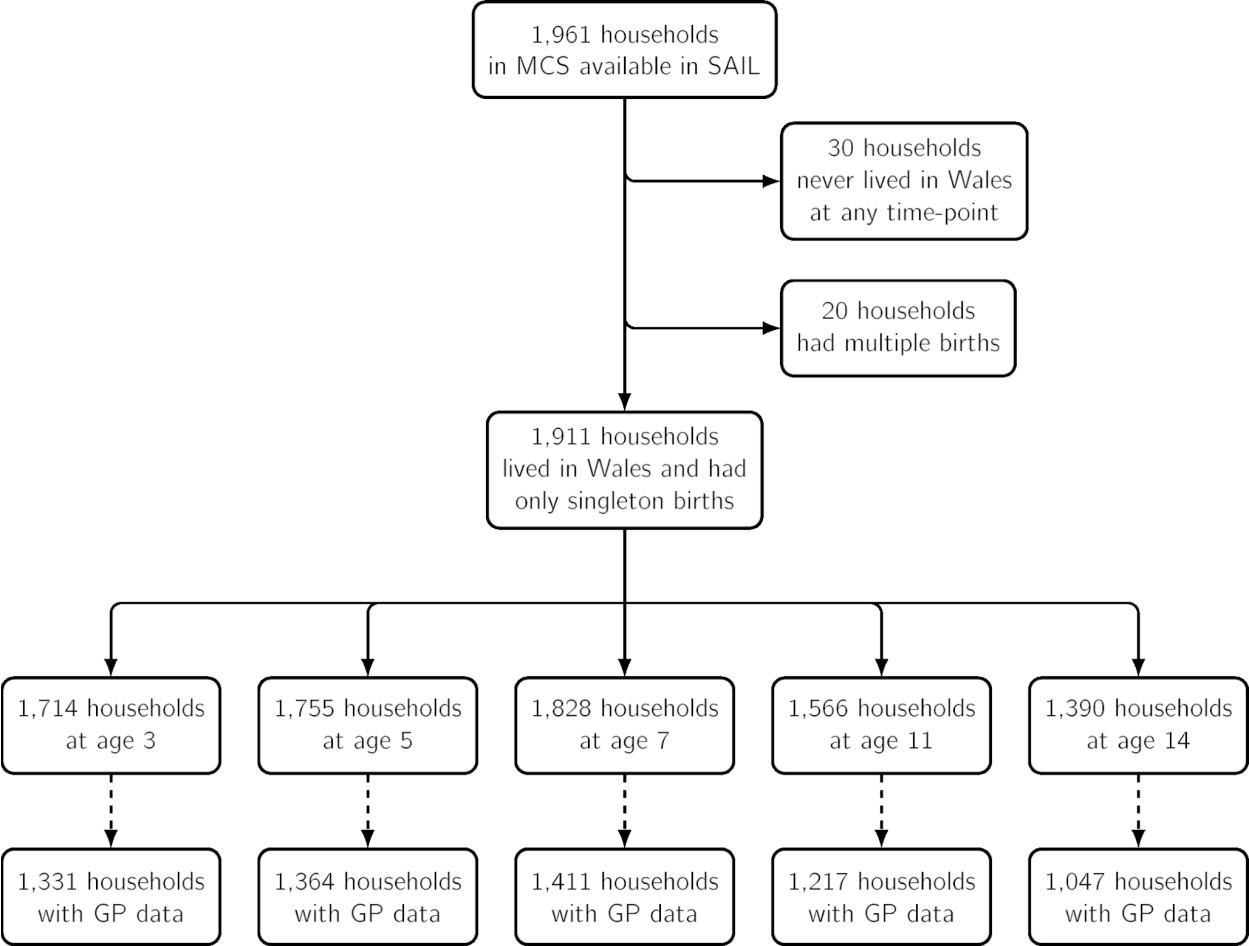
Sample selection households available given time-point and data linkage availability

We linked data on Welsh-domiciled families from the MCS (Connelly & Platt, 2014) with administrative data from maternal mental health General Practice (GP) records. MCS sampled a cohort of all children born between 1st September 2000 and 31st August 2001 in Wales, with surveys (to date) at seven different time points (a child aged 0, 3, 4, 7, 11, 14 and 17 years old). To be included in the initial cohort, children had to be eligible to receive Child Benefit, a form of state benefit in the UK (Claim Child Benefit, 2021). The Welsh subgroup of the MCS was stratified by socioeconomic status, which over-sampled children residing in the economically poorest areas (ward-based Child Poverty Index) (Plewis, 2007). The families’ consent was obtained when the children were aged seven years old to link survey data to electronic health record (EHR) data sources, permitted up to the child’s 14th birthday (Tingay et al., 2019). The data were accessed via the SAIL Databank, a Trusted Research Environment (TRE) in Wales for the 90.7% of families who consented to data linkage and approved through rigorous governance procedures (for further description of the de-identified data linkage procedures of MCS to health records, see Tingay et al. (2019)). We used probabilistic matching for more successful matches and higher quality. Figure 1 shows the sample selection process. We removed all households who never lived in Wales at any sweep and multiple births (i.e., twins), giving a linkage to 1,911 households, with linkage to GP data causing variation in the sample size across the MCS survey sweeps. We accessed the data following Information Governance Review Panel (IGRP) approval, which permits access to anonymised data via SAIL Databank.

### Measures

#### Maternal Mental Health

**Fig. 2:**
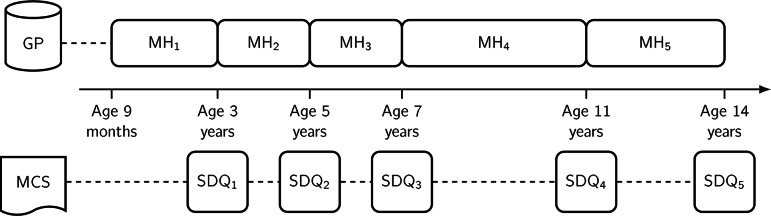
Time periods of data collection for maternal mental health (MH_1-5_) and child strengths and difficulties (SDQ_1 – 5_)

Validated code lists for common mental health disorders (John et al., 2016) were used to select individuals who present with symptoms of, or are diagnosed or treated for, depression and anxiety. In Fig. 2, the time periods of each measure are shown; for example, maternal mental health at time-point 1 was any event between the child being aged 9 months old and 3 years old.

### Child Problem Behaviours

Measures of child problem behaviours were taken from the MCS parent-reported data. We used the Strengths and Difficulties Questionnaire (SDQ), a brief emotional and behavioural screening questionnaire, capturing children’s emotional, behavioural, hyperactivity or peer problems, with satisfactory reliability across internal consistency (α: 0.73) and cross-informant correlation (mean 0.34) (Goodman, 2001). A total SDQ score (derived from summing responses from the four subscales of emotional, conduct and peer problems along with hyperactivity) can be categorised into close to average, slightly raised, high and severe as per guidance (EHCAP, 2014). We analysed five SDQ outcomes of total strength and difficulties score, emotional problems, conduct problems, hyperactivity, and peer problems.

### Demographics

We explored our sample distributions in terms of child sex (female/male), birth weight, gestational age, breastfeeding, family income and mothers’ highest academic and/or vocational qualification whereby a higher qualification indicates more educational qualifications (‘None’, “NVQ1 or GCSE D-G’, ‘NVQ2 or Apprenticeship or GCSE A* - C’, ‘NVQ3 or A-level’, ‘Professional qualification or Degree or Diploma or Health professional’, ‘Higher degree’); these measures were taken when the child was 9 months of age.

### Data Analysis

We managed, cleaned, and conducted descriptive analysis in Stata 17 (StataCorp, 2021) and performed structural equation modelling in Mplus version 8.7 (Muthén & Muthén 2017). We specified five random intercept cross-lagged panel models (RI-CLPM) to analyse the longitudinal panel data for each child problem behaviour. The RI-CLPMs’ temporal and concurrent associations between maternal mental health and children’s problem behaviours were explored within individuals across the five time points shown in Fig. 2, an approach used by Kristensen et al. (2021). As Mund and Nestler (2019) discussed, the RI-CLPM permits each individual to fluctuate around their own stable, trait-like level over time with respect to the other variables in the model. This method was selected over the cross-lagged panel model (CLPM), which Hamaker et al. (2015) critiqued as an erroneous method to represent lagged estimates of within-person relationships over time.

We estimated these models using a Bayesian approach, and followed the new methodological guidance provided by Mplus, where categorical RI-CLPM are estimated by residuals (Asparouhov & Muthen, 2021). In conducting the models, we used non-informative prior distributions and ensured the Posterior Predictive Check had intersected the null, and the p-value was > 0.05 (Asparouhov & Muthén, 2021). Likewise, we ensured the Proportional Scale Reduction (PSR) value evidenced a downward trend per iteration and that values were largely < 1.01 for good convergence. We visually inspected trace plots for all parameters to assess non-convergence (Muthén, 2010). If there appeared to be evidence of non-convergence, we increased the number of iterations and then thinned our models (selection of separated points at each k-th step) by 10 or 20. The Bayesian estimator in Mplus uses Full Information Maximum Likelihood to account for missing data; however, our scores of SDQ use listwise deletion in that all items must have a response to be included and non-response is greater in total SDQ compared to subscales (see Supplementary Material Table 1). To date, frequentist approaches do not yet offer the same techniques for missing data; the Bayesian approach was selected given the flexible framework and ability to account for uncertainty in estimates.

## Results

There were more boys in the sample (52%), most children had a normal birth weight (90%), and most had a gestational age above 36 weeks (~ 95%). The most common qualification among mothers was GCSE A* - C (or equivalent), and the most common family net income band was £10,400 - £20,800 (32%). The baseline demographics of the children in the MCS cohort at time-point 3 (N = 1,828) compared to the sample of mothers who had GP data available at the same time-point (N = 1,411) were very similar, see Supplementary Material Table 2.

### Random Intercept Cross-lagged Panel Models

**Fig. 3:**
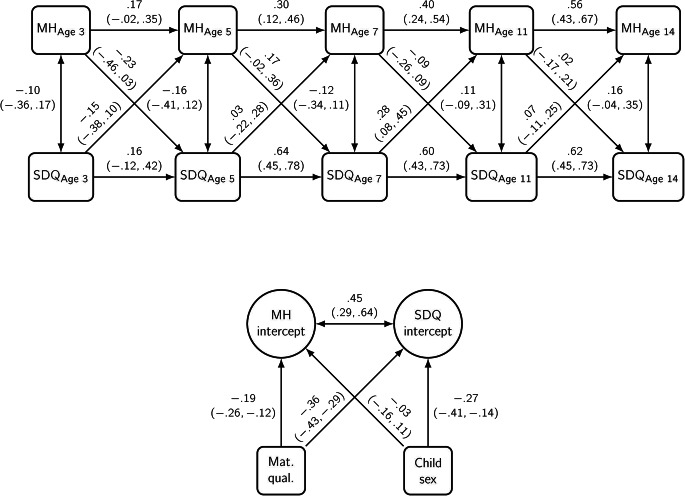
Probit estimates with 95% credible intervals from a random intercept cross-lagged panel model of total Strengths and Difficulties Questionnaire (SDQ) score and maternal mental health (MH). *Adjusted for maternal qualifications and child sex (N = 1835)

**Fig. 4:**
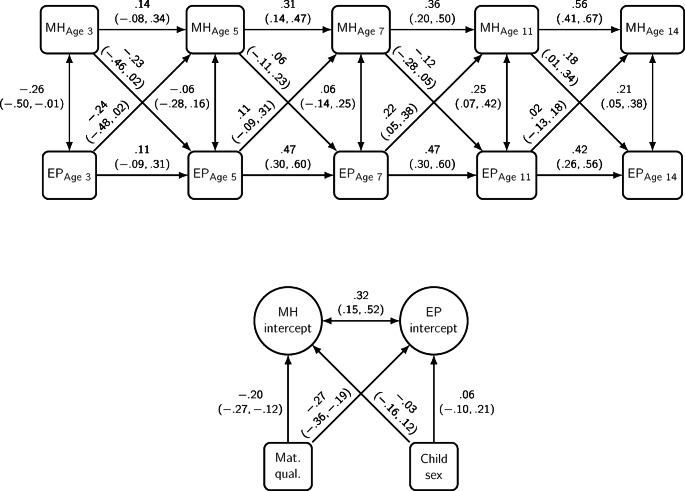
Probit estimates with 95% credible intervals from a random intercept cross-lagged panel model of Emotional Problems (EP) and maternal mental health (MH). *Adjusted for maternal qualifications and child sex. (N = 1842)

**Fig. 5:**
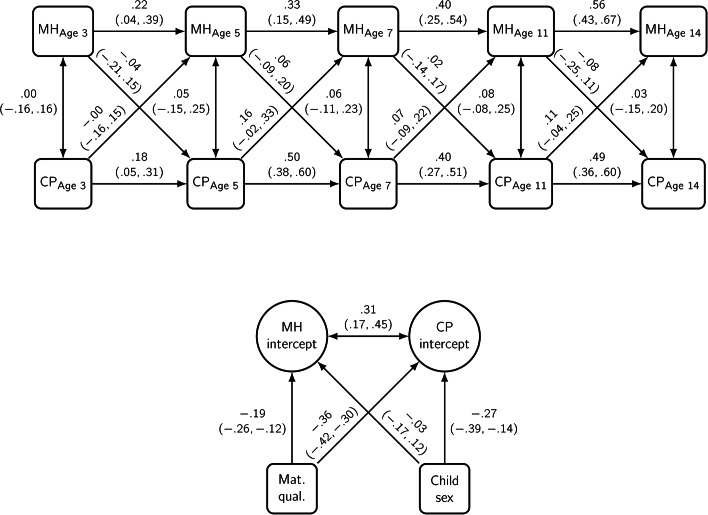
Probit estimates with 95% credible intervals from a random intercept cross-lagged panel model of Conduct Problems (CP) and maternal mental health (MH). *Adjusted for maternal qualifications and child sex. (N=1842)

Five RI-CLPMs estimated the relationship between maternal mental health (MH) and total SDQ (Fig. 3), emotional problems (EP) (Fig. 4), conduct problems (CP) (Fig. 5), hyperactivity (HYP) and peer problems (PPR), adjusted for maternal qualifications and child sex. A positive coefficient represents an increase in the predicted probability as the predictor increases, and a negative coefficient represents a decrease in the predicted probability as the predictor increases; 95% Credible Intervals (CI) are given in brackets. Throughout the models, maternal mental health (MH) had positive carry-over stability effects across time, which suggests that help-seeking behaviours, compared to their own norm, were likely to follow during the next period. Likewise, all types of child problems had positive carry-over stability effects, except for time-point 1.

### Total Strengths and Difficulties Score

For cross-lagged effects, SDQ_Age 7_ was associated with increased maternal mental health problems at MH_Age 11_ (0.28, 95%CI 0.08, 0.45), see Fig. 3. The between-person level correlation showed a strong, positive association suggesting both had similar trait-like difficulties simultaneously (0.45, 95%CI 0.29, 0.64). The correlation between mother’s mental health problem and child SDQ cross-sectionally had no associations. Child sex was not associated with maternal mental health (-0.03, 95%CI − 0.16, 0.11), but girls were at a lower risk of problem behaviours (-0.27, 95%CI − 0.41, − 0.14). Higher maternal qualifications were associated with reduced risk for both maternal mental health (-0.19, 95%CI − 0.26, − 0.12) and child problem behaviours (-0.36, 95%CI − 0.43, − 0.29).

### Emotional Problems

For cross-lagged effects, there was a positive association from EP_Age 7_ to MH_Age 11_ (0.22, 95%CI 0.05, 0.38), and then MH_Age 11_ was positively associated with EP_Age 14_ (0.18, 95%CI 0.01, 0.34); see Fig. 4. The between-person level correlation showed a strong, positive association suggesting both had similar trait-like difficulties simultaneously (0.32, 95%CI 0.15, 0.52). The correlation between maternal mental health problems and child emotional problems cross-sectionally showed narrowing positive credible intervals over time and associations were clearer at Age 11 (0.25, 95%CI 0.07, 0.42) and Age 14 (0.21, 95%CI 0.05, 0.38). Child sex was not associated with maternal mental health (-0.03, 95%CI − 0.16, 0.12), or emotional problems (0.06, 95%CI − 0.10, 0.21). Higher maternal qualifications were associated with reduced risk for both maternal mental health (-0.20, 95%CI − 0.27, − 0.12) and child emotional problems (-0.27, 95%CI − 0.36, − 0.19).

### Conduct Problems

For cross-lagged effects, maternal mental health (MH) had wide credible intervals with no clear relationship with conduct problems (CP) (see Fig. 5). The between-person level correlation showed a strong, positive association suggesting both had similar trait-like difficulties simultaneously (0.31, 95%CI 0.17, 0.45). The correlation between maternal mental health and conduct problems cross-sectionally had no apparent relationship. Child sex was not associated with maternal mental health (-0.03, 95%CI − 0.17, 0.12) but was strongly associated with conduct problems (-0.27, 95%CI − 0.39, − 0.14). Higher maternal qualifications were associated with reduced risk for both maternal mental health (-0.19, 95%CI − 0.26, − 0.12) and child problems (-0.36, 95%CI − 0.42, − 0.30).

### Hyperactivity

For cross-lagged effects, maternal mental health (MH) had wide credible intervals with no clear relationship with child hyperactivity (HYP). Likewise, hyperactivity had no clear relationship with maternal mental health. The between-person level correlation showed a strong, positive association suggesting both had similar trait-like difficulties simultaneously (0.33, 95%CI 0.20, 0.46). The correlation between maternal mental health problem and hyperactivity cross-sectionally had no association, aside from at Age 14, which was positive (0.26, 95%CI 0.08, 0.44). Child sex was not associated with maternal mental health (-0.02, 95%CI − 0.16, 0.12), but girls were at a lower risk of hyperactivity (-0.45, 95%CI − 0.57, − 0.33). Higher maternal qualifications were associated with reduced risk for both maternal mental health (-0.19, 95%CI − 0.26, − 0.12) and hyperactivity (-0.28, 95%CI − 0.34, − 0.22). See Supplementary Material Fig. 1.

### Peer Problems

For cross-lagged effects, maternal mental health (MH) had wide credible intervals with no clear relationship with peer problems (PPR). However, PPR_Age 7_ had a positive association with MH_Age 11_ (0.25, 95%CI 0.09, 0.39); all other time points from PPR to MH showed no associations and wide credible intervals. The between-person level correlation showed a strong, positive association suggesting both had similar trait-like difficulties simultaneously (0.32, 95%CI 0.13, 0.50). The correlation between maternal mental health problem and peer problems cross-sectionally had a negative relationship in the first time-point (-0.26, 95%CI − 0.47, − 0.04), however, age 11 had a positive relationship (0.28, 95%CI 0.12, 0.44). Child sex was not associated with maternal mental health (-0.02, 95%CI − 0.16, 0.12), but girls were at a lower risk of peer problems (-0.23, 95%CI − 0.39, − 0.08). Higher maternal qualifications were associated with reduced risk for both maternal mental health (-0.19, 95%CI − 0.26, − 0.12) and peer problems (-0.33, 95%CI − 0.42, − 0.25). See Supplementary Material Fig. 2.

## Discussion

In this study, we investigated the longitudinal association between maternal mental health problems and children’s emotional, behavioural, hyperactivity, and peer problems. We found mixed evidence for bi-directional relationships between maternal mental health and child problem behaviours. Our findings may have implications for theory advancement and intervention strategies aimed at improving maternal mental health and children’s problem behaviours.

### Trait-like Associations (Between-person)

In support of hypothesis one, our research consistently showed strong between-person (trait-like) associations between maternal mental health and child problems, whereby mothers with mental health problems likely reported that their children had problem behaviours throughout childhood and into adolescence. We consistently found evidence for socioeconomic status (via early maternal education qualifications) being strongly associated with both maternal and child problems, in line with the general literature (Finegan et al., 2018; Letourneau et al., 2013; Marmot & Bell, 2012; Reiss, 2013). Given mothers with fewer resources are at a greater risk of mental health problems (depression and anxiety), which subsequently is associated with less desirable parenting practices and styles (Vreeland et al., 2019), it is plausible to advocate that mothers who are ‘doubly-disadvantaged’ should be a priority for support. Other studies have also referred to between-effects being in relation to lower sensitivity from the mother to the child (Murray et al., 1996), communication styles (Murray & Cooper, 1997), family conflict or monitoring (Van Loon et al., 2014) and genetic transmission.

We also found that boys were at a heightened risk of overall problems, including conduct, hyperactivity, and peer problems, mirroring findings by Maurice-Stam et al. (2018) and also Van Roy et al. (2006). Contrastingly, we did not find that emotional problems were higher for girls unlike other studies (Bøe et al., 2016; Van Roy et al., 2006). However, wider studies suggest that emotional problems in girls tend to have adolescent on-set which may explain our study’s lack of alignment (Martel, 2013; Murray et al., 2022; Rutter et al., 2003). In terms of hyperactivity, our findings generally align with boys having a greater prevalence than girls (Williamson & Johnston, 2015). However, ongoing research suggests that measures of ADHD could be biased towards symptoms which align more with boys’ experiences (Williamson & Johnston, 2015), with ongoing research on how to better identify girls’ symptoms and experiences (Young et al., 2020). In terms of the measurement performance longitudinally, Murray et al. (2022) found that the parent-reported SDQ items did not perform well as a latent variable at age three for males and females, which may explain why fewer associations were observed at this age; this study uses the same data, but we use the Welsh sub-group to derive estimates.

As a result, given both the mother and child experienced problems, we echo Ranøyen et al. (2015) that interventions should be family-based to both evaluate and support all members of the household, as demonstrated in an intervention by Poole et al. (2018). However, support strategies should be aware of both the socioeconomic vulnerability of families who may require economic support, and sex differences, with boys being at a higher risk for problem behaviour(s).

### Carry-over Stability Effects (Within-person)

In support of hypothesis two, all models showed consistent within-person carry-over stability effects, suggesting earlier child problems increased the likelihood of later problems, congruent with the literature on child development (Flouri et al., 2015; Sifaki et al., 2021) and other bi-directional studies (Belsky, 1984; Speyer et al., 2022; Xerxa et al., 2021). Carry-over stability effects became stronger over time for maternal mental health, with associations increasing in size at each time-point, whereas associations for child problems were more stable, but earlier time points were weaker, suggesting age five years may be a critical point for the development of problems. Our within-person results are in line with previous research on maternal mental health in earlier post-partum stages (Horowitz & Goodman, 2004) and child behaviour problems (Gross et al., 2008). Furthermore, our results for child within-associations closely resemble results found in Speyer et al. (2022), which is expected given they used the same dataset but this was UK-wide, not the Wales sub-sample like our study.

### Bi-directional Associations Over Time

We found mixed results for bi-directional associations, with the differences in findings depending on the problem behaviour outcome of the child. To summarise, child emotional problems appeared to be the only outcome which had some evidence of bi-directional associations occurring in mid-childhood to mid-adolescence (child aged 7–14 years). Our findings regarding children’s emotional problems are supported by the transactional theory literature (Bell, 1968, 1971; Belsky, 1984; Sameroff, 1975), the premise that children are influenced by their environment while simultaneously responding to the environment they are in (Cicchetti & Toth, 1997). As discussed by Speyer et al. (2022), maternal mental health problems can lead to the use of parenting behaviours and styles that can subsequently lead to child problems, which then cycle back to the mother through the child’s behaviour. Research has previously evidenced the reciprocal relationships in the domain of child behaviour, in terms of parental depression (Gross et al., 2008) or mental health (Speyer et al., 2022; Xerxa et al., 2021), but also parenting stress which has been discussed as an antecedent and a consequence of child behaviour problems (Neece et al., 2012; Vallotton et al., 2016).

Given that child emotional problems show bi-directional associations in both directions, i.e., child problems associating with maternal problems and then vice-versa, our findings support the theory that children and their mother mutually affect one another, eliciting specific responses from each other (Sameroff, 1975; Xerxa et al., 2021). We see that child emotional problems at age 7 years were initially associated with maternal mental health, and reciprocally, maternal mental health then was associated with child emotional problems at age 14 years. Speyer et al. (2022) also found a reciprocal relationship for emotional problems, however, this occurred earlier, started with the mothers’ association first, and only occurred for boys. Consequently, we observe that reciprocal relationships are likely to exist but the directions in which they occur require further investigation with a range of samples and measurements to derive comparisons.

We also find that the timing of these associations is of interest. Mid to late stages of childhood and early adolescence held reciprocal associations rather than in the early years. Our study does not have a further time-point to investigate later adolescence at the time of writing, but in Speyer et al. (2022) which used similar methods and data, associations were continued to age 17 years. While it is often the case that the life phase of adolescence is depicted by the ability to simultaneously become influenced and influence peers more so than parents (Brechwald & Prinstein, 2011; Collins & Laursen, 2004), our findings and other studies still find an association in adolescence. This is perhaps due to the parent-child relationship not ceasing but changing whereby parental closeness often becomes second to friends (Collins & Laursen, 2004; Laursen & Williams, 1997). Other studies have also identified reciprocal relationships in the area of emotional problems at ages 7 to 14 years (Serbin et al., 2015; Speyer et al., 2022; Xerxa et al., 2021). Hence, our study confirms that bi-directional research should consider the childhood and adolescent life phase as timings appear to be important and may change.

In terms of the other problem behaviour areas, we did not find bi-directional associations for child conduct problems, and we found little to no evidence for bi-directional associations in the areas of child hyperactivity and peer problems. We discuss each child’s problem behaviour outcome in relation to wider theory, literature, and practice below.

#### Children’s Overall Problems and Maternal Mental Health

For overall problems (total SDQ score), we found some support for hypothesis four, that child overall problems were positively associated with maternal mental health problems when the child was 7 – 11 years old. All other associations intersected the null, suggesting uncertainty in estimates. Child overall problem behaviour in this study represents a constellation of behaviours (emotional, conduct, hyperactivity, and peer problems) where levels are not ‘typical’ across these items. It is well-known that parenthood is a substantial life change which can be stressful, particularly if children are diagnosed with physical or mental disabilities (Hayes & Watson, 2013). Developmental transition periods such as middle childhood can be of particular importance for child driven-effects (Blume et al., 2022; Yan & Ansari, 2016). Jiang et al. (2022) found that parenting stress was largely predicted by child problem behaviours, however, there was a reciprocal association in early childhood, with internalising behaviour being specifically larger in effect size and more prevalent over time compared to externalising in disadvantaged families (a similar cohort to our study in some aspects). Dubois-Comtois et al. (2013) explain that aspects such as the mother-child relationship can be key for promoting child adaptation, along with secure attachment styles; children who had a controlling-punitive attachment to their caregivers were more likely to show socio-emotional difficulties. Hence, to understand the pathways of child problem behaviours and maternal mental health further, research must explore the multiplicity of the parent-child relationship including factors such as attachment, parenting, the family environment, and social support.

#### Children’s Emotional Problems and Maternal Mental Health

We found that children’s emotional problems at age 7 years were positively associated with maternal mental health problems when the child was aged 11 years. Reciprocally, maternal mental health problems when the child was 11 years were positively associated with child emotional problems at age 14. While it is not clear why emotional problems were elevated at age 7 for children, it appears that they may disrupt maternal mental health, which then later reinforces emotional problems, which builds on the current evidence base (Ranøyen et al., 2015; Speyer et al., 2022; Turney, 2012; Xerxa et al., 2021) and transactional theory (Bell, 1968, 1971; Belsky, 1984; Sameroff, 2009).

In terms of explanations, an insecure attachment was identified as a mechanism between maternal mental health and child internalising symptoms in a meta-analysis (Spruit et al., 2020); a moderate effect size was found *r* = .31 from 123 samples. Secure attachment is considered met when a child views their parent as a ‘secure base’ alleviating fear, stress and worry (Bowlby, 2012); mothers with symptoms of depression are at a higher risk for insecure attachment, which is related to the child having an increased risk of dysfunctional cognitions about the self and chronic stress (due to a lack of a secure base for alleviation) (Spruit et al., 2020). In a transactional sense, it could be argued that depressive symptoms not only may change behaviour, (i.e., compromised parenting behaviours (Vreeland et al., 2019)) but also how emotions are viewed and felt by the parent (positive affect) (Field et al., 1988). This may lead to the child developing similar behaviours that could be both environmental and genotype-specific (Hyde et al., 2016), which then may reinforce the mother’s emotional climate and behaviours, essentially curating a cycle.

#### Children’s Conduct Problems and Maternal Mental Health

We found no evidence that maternal mental health problems were associated with child conduct problems. Our study’s lack of alignment with other research was not expected. Particularly given other studies have suggested a bi-directional link between parental depressive symptoms and child externalising behaviour (Gross et al., 2008), and the coercive family process model describes how detrimental family interactions can initiate and maintain behavioural problems in children (Patterson, 2002b). Gross et al. (2008) found strong evidence for bi-directional relationships whereby both the mother and child increased the risk of problem behaviours or depressive symptoms across two reports or raters. The difference may be related to administrative data being used which can sometimes signify a more serious illness, or be biased toward those who can identify a problem or seek help (Davis et al., 2016), whereas Gross et al. (2008) used continuous self-reported measures which have more variability and do not depend on participants attending healthcare providers.

#### Children’s Hyperactivity and Maternal Mental Health

We found no evidence that hyperactivity and maternal mental health problems were associated. Still, we found a cross-sectional correlation at age 14 years, which may be due to the increased demand on the parent as the child becomes more autonomous. As discussed, providing care for a child who has hyperactivity can place additional demands and mental challenges on the parent (Johnston & Mash, 2001), which could become a mental health problem. Unlike our study, Breaux and Harvey (2019) found bi-directional associations between child ADHD symptoms and maternal depression. They theorise that this association is explained by mechanisms of parenting and family functioning, whereby increased ADHD symptoms placed additional demands on the parent, and a potential response was negative parenting practices (Barkley et al., 1991). However, in this study we did not find evidence of this, which may be attributable to (1) the use of administrative data, or (2) the focus on hyperactivity than specific ADHD diagnoses, which may capture more demanding behaviour.

#### Children’s Peer Problems and Maternal Mental Health

We found no association between maternal mental health and peer problems over time, but an association between peer problems at age 7 years and maternal mental health problems when the child was 11 years old. These findings may allude to increased pressure on the parent when the child’s social skills are more evident, and while studies have suggested it occurring in the opposite direction (Waerden et al., 2015), other researchers have found evidence of reciprocal relationships. For instance, Sifaki et al. (2021) used a cross-lagged panel model and found bi-directional relationships between paternal distress and peer problems. They theorise that peer victimisation or bullying could lead to feelings of shortcoming as a parent, or a source for further concern (Sifaki et al., 2021). Moreover, our theoretical thinking resides in that caregivers with greater depressive or anxiety symptoms are more likely to use parenting styles or behaviours which are considered less effective for child development in some cultural contexts. Yamagata et al. (2013) found bi-directional effects between parenting and peer problems in childhood; mechanisms in parental strain or behaviours may explain the association found in this study, however, bi-directional associations were not identified over time. As such, there may be critical points in childhood or specific areas of emotional mood rather than mental illness which require further investigation.

### Strengths and Limitations

Our study benefits from several strengths, specifically the linkage of rich, detailed cohort data with routinely collected data over 13 years. Moreover, our method allowed for the separating of within and between-person effects to isolate bi-directional associations and benefits from a powerful new Bayesian approach. However, we could not access data on prenatal maternal mental health, despite research suggesting that it is associated with long-term development outcomes in children via elevated hormones and stress (Lupien et al., 2009; Welberg & Seckl, 2001). Second, we recognise that our study may be liable to ‘shared variance’ as discussed by Xerxa et al. (2021). Shared variance is a form of information bias where one or more participants reports on multiple aspects of the study, for example, a mother reporting on their own mental health and then their child’s (Xerxa et al., 2021). Collishaw et al. (2009) found that all correlates of child mental health were related differently to parent, teacher and child ratings, with parent-ratings of both their functioning and child functioning being the most strongly correlated. Indeed, it is known that mothers experiencing mental health illness may report higher child behavioural problems, whereas mothers not experiencing mental illness tend not to report child behavioural problems as much (Najman et al., 2000; Ringoot et al., 2015). In addition, if an external factor influences both the rating of the mother and child this can also affect the results (Xerxa et al., 2021). While we use administrative measures of mothers’ mental health symptoms, treatment and diagnoses, the child behaviours are largely reported by the mother whose rating may be affected by their own symptoms. Hence, further studies should consider a multitude of reporting (administrative and self) by various participants (partners, teachers, practitioners, and children themselves) to fully understand the consequences of shared method variance.

Third, by not including fathers or partners in this study, it is not possible to adjust for potential areas of strain or support. In addition, we also did not test for possible mediators or mechanisms in this research, so cannot empirically determine this in our study. Fifth, we are reliant on individuals accessing healthcare systems, reporting problems and outcomes being correctly coded by GPs, so our figures may be underestimated. Moreover, our sample over-represents economically disadvantaged groups, who are more likely to experience mental health problems (Reiss, 2013). Our study is likely to be more representative of these groups, rather than the total population, or families with more resources (i.e., higher socioeconomic status). Lastly, healthcare utilisation is more likely to depict feelings of ‘readiness’ towards receiving treatment which differs from self-reported mood.

## Conclusion

Overall, our study using linked survey and administrative data found mixed evidence to support bi-directional theories of development (Bell, 1968, 1971). Child emotional problems had bi-directional associations in mid to late childhood, whereas overall behavioural problems and peer problems were associated only in the direction from child problems to maternal mental health. On the other hand, no cross-lagged associations were found for conduct problems or hyperactivity. However, all models had strong between-person effects suggesting other aspects, such as socioeconomic status, were largely explanatory for the relationship between maternal mental health and child problems. We found that lower socioeconomic groups were at a greater risk for both maternal mental health problems and child problems, and boys were particularly at risk for all domains except emotional problems. Therefore, we strongly encourage policymakers to provide family-based support for caregivers and children experiencing difficulties in terms of mental health, child behaviours or parenting, which considers the economic vulnerability that some families may experience.

### Electronic Supplementary Material

Below is the link to the electronic supplementary material.

**Supplementary material: Table S1**: Frequencies of maternal mental health and child difficulties at each time-point. **Table S2**: Baseline demographics of children in the total MCS cohort and the GP registered sample. Due to disclosure risks with sample counts between 1 and 4, these have been masked as ‘#x003C;5’ and neighbouring categories rounded to the nearest 5 and marked as (~N, ~%). **Figure S1**: Probit estimates with 95% credible intervals from a random intercept cross-lagged panel model of Hyperactivity (HYP) and maternal mental health (MH). *Adjusted for maternal qualifications and child sex. (N=1841). **Figure S2**: Probit estimates with 95% credible intervals from a random intercept cross-lagged panel model of Peer Problems (PPR) and maternal mental health (MH). *Adjusted for maternal qualifications and child sex. (N=1838).

## Data Availability

The data used in this study are available in the SAIL Databank at Swansea University, Swansea, UK, but as restrictions apply, they are not publicly available. All proposals to use SAIL data are subject to review by an independent Information Governance Review Panel (IGRP). Before any data can be accessed, approval must be given by the IGRP. The IGRP gives careful consideration to each project to ensure proper and appropriate use of SAIL data. When access has been granted, it is gained through a privacy protecting safe haven and remote access system referred to as the SAIL Gateway. SAIL has established an application process to be followed by anyone who would like to access data via SAIL at https://www.saildatabank.com/application-process All research conducted has been completed under the permission and approval of the SAIL independent IGRP project number 1015.
