# Supplementary material for: Maternal Mental Health and Children’s Problem Behaviours: A Bi-directional Relationship?
Source: Res Child Adolesc Psychopathol. 2023 Jul 4;51(11):1611–26. doi: 10.1007/s10802-023-01086-5 (PMC10628040; doi:10.1007/s10802-023-01086-5)
Supplement: Supplementary file 1 — Supplementary material: Table S1: Frequencies of maternal mental health and child difficulties at each time-point. Table S2: Baseline demographics of children in the total MCS cohort and the GP registered sample. Due to disclosure risks with sample counts between 1 and 4, these have been masked as ‘#x003C;5’ and neighbouring categories rounded to the nearest 5 and marked as (~N, ~%). Figure S1: Probit estimates with 95% credible intervals from a random intercept cross-lagged panel model of Hyperactivity (HYP) and maternal mental health (MH). *Adjusted for maternal qualifications and child sex. (N=1841). Figure S2: Probit estimates with 95% credible intervals from a random intercept cross-lagged panel model of Peer Problems (PPR) and maternal mental health (MH). *Adjusted for maternal qualifications and child sex. (N=1838). [file 10802_2023_1086_MOESM1_ESM.docx]

**Supplementary material**

| **Mother’s mental health** | | | | | |
| --- | --- | --- | --- | --- | --- |
|  | **Time-point 1** | **Time-point 2** | **Time-point 3** | **Time-point 4** | **Time-point 5** |
| No event | 947 (71%) | 1018 (75%) | 1012 (72%) | 683 (56%) | 718 (69%) |
| MH event | 384 (29%) | 346 (25%) | 399 (28%) | 534 (44%) | 329 (31%) |
| Total | 1331 (100%) | 1364 (100%) | 1411 (100%) | 1217 (100%) | 1047 (100%) |
| **Total SDQ** | | | | | |
| Close to average | 981 (83%) | 1270 (90%) | 1360 (89%) | 1255 (88%) | 1062 (85%) |
| Slightly raised | 116 (10%) | 79 (6%) | 68 (5%) | 84 (6%) | 75 (6%) |
| High | 48 (4%) | 34 (2%) | 54 (4%) | 44 (3%) | 50 (4%) |
| Severe | 37 (3%) | 32 (2%) | 42 (3%) | 47 (3%) | 62 (5%) |
| Total | 1182 (100%) | 1415 (100%) | 1524 (100%) | 1430 (100%) | 1249 (100%) |
| **Emotional sub-scale** | | | | | |
| Close to average | 1438 (92%) | 1510 (91%) | 1533 (88%) | 1239 (82%) | 1018 (78%) |
| Slightly raised | 73 (5%) | 79 (5%) | 93 (5%) | 111 (7%) | 93 (7%) |
| High | 37 (2%) | 60 (3%) | 87 (5%) | 108 (7%) | 108 (8%) |
| Severe | 9 (1%) | 14 (1%) | 31 (2%) | 53 (4%) | 82 (6%) |
| Total | 1557 (100%) | 1663 (100%) | 1744 (100%) | 1511 (100%) | 1301 (100%) |
| **Conduct sub-scale** | | | | | |
| Close to average | 779 (49%) | 1315 (79%) | 1414 (80%) | 1195 (79%) | 1060 (81%) |
| Slightly raised | 266 (17%) | 196 (12%) | 179 (10%) | 173 (12%) | 121 (9%) |
| High | 362 (23%) | 128 (8%) | 130 (7%) | 114 (8%) | 92 (7%) |
| Severe | 167 (11%) | 35 (2%) | 34 (2%) | 23 (2%) | 32 (2%) |
| Total | 1574 (100%) | 1674 (100%) | 1757 (100%) | 1505 (100%) | 1305 (100%) |
| **Hyperactivity sub-scale** | | | | | |
| Close to average | 1121 (75%) | 1332 (82%) | 1390 (79%) | 1240 (82%) | 1104 (85%) |
| Slightly raised | 240 (16%) | 193 (12%) | 193 (11%) | 157 (10%) | 117 (%) |
| High | 66 (4%) | 43 (3%) | 76 (4%) | 46 (3%) | 40 (3%) |
| Severe | 58 (4%) | 65 (3%) | 90 (5%) | 63 (4%) | 45 (4%) |
| Total | 1485 (100%) | 1633 (100%) | 1749 (100%) | 1506 (100%) | 1306 (100%) |
| **Peer problem sub-scale** | | | | | |
| Close to average | 1130 (82%) | 1330 (87%) | 1387 (86%) | 1197 (81%) | 979 (75%) |
| Slightly raised | 132 (10%) | 99 (6%) | 115 (7%) | 118 (8%) | 126 (10%) |
| High | 78 (6%) | 55 (4%) | 68 (4%) | 80 (5%) | 81 (6%) |
| Severe | 37 (3%) | 48 (3%) | 51 (3%) | 82 (6%) | 120 (9%) |
| Total | 1377 (100%) | 1532 (100%) | 1621 (100%) | 1477 (100%) | 1306 (100%) |

Table S1: Frequencies of maternal mental health and child difficulties at each time-point

|  | **MCS Welsh sample at time-point 3** **(N=1828)** | **GP registration at time-point 3** **(N=1411)** |
| --- | --- | --- |
| **Child sex** | | |
| Male | 946 (52%) | 739 (52%) |
| Female | 882 (48%) | 672 (48%) |
| Missing | - | - |
| **Birthweight** | | |
| Low | ~210 (~10%) | ~165 (~10%) |
| Normal | ~1,620 (~90%) | ~1,250 (~90%) |
| Missing | <5 | <5 |
| **Breastfeeding** | | |
| Tried to breastfeed | 1,140 (~60%) | ~880 (~60%) |
| Did not try | 690 (~40%) | ~535 (~40%) |
| Missing | <5 | <5 |
| **Mother's highest qualification** | | |
| None | 247 (14%) | 198 (14%) |
| NVQ1/GCSE D-G | 325 (18%) | 239 (17%) |
| NVQ2/Apprenticeship/GCSE A* -C | 673 (37%) | 529 (37%) |
| NVQ3/A-level | 174 (10%) | 135 (10%) |
| Professional qualification/Degree/Diploma/Health professional | 356 (20%) | 272 (19%) |
| Higher degree | 22 (1%) | 13 (1%) |
| Missing | 31 (2%) | 25 (2%) |
| **Family net income** | | |
| £0 – 3099 | 25 (1%) | 19 (1%) |
| £3100 – 10400 | 485 (27%) | 375 (27%) |
| £10400 – 20800 | 601 (32%) | 470 (33%) |
| £20800 – 31200 | 351 (19%) | 266 (19%) |
| £31200 – 52000 | 226 (12%) | 182 (13%) |
| £52000+ | 61 (3%) | 42 (3%) |
| Missing | 79 (4%) | 57 (4%) |
| **Gestational age** | | |
| 25 – 28 weeks | <5 | <5 |
| > 28 – 32 weeks | ~10 (<5%) | ~10 (~1%) |
| > 32 – 36 weeks | ~70 (~5%) | ~55 (~5%) |
| > 36 – 40 weeks | ~950 (~50%) | ~720 (~50%) |
| > 40 – 43 weeks | ~780 (~45%) | ~615 (~45%) |
| Missing | ~5% | ~15 (~1%) |

Table S2: Baseline demographics of children in the total MCS cohort and the GP registered sample. Due to disclosure risks with sample counts between 1 and 4, these have been masked as '<5' and neighbouring categories rounded to the nearest 5 and marked as (~N, ~%)


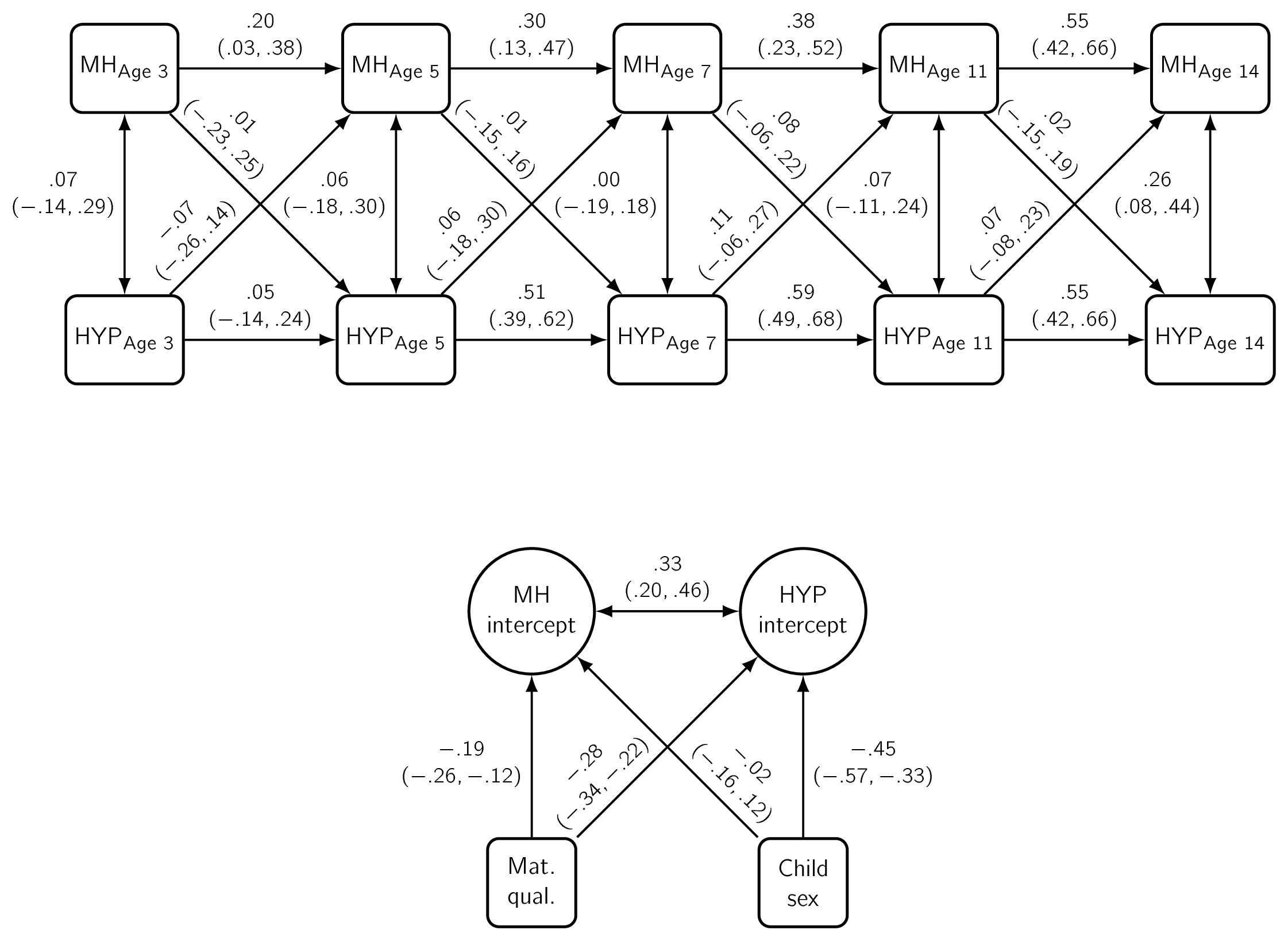


Figure S1: Probit estimates with 95% credible intervals from a random intercept cross-lagged panel model of Hyperactivity (HYP) and maternal mental health (MH). *Adjusted for maternal qualifications and child sex. (N=1841)


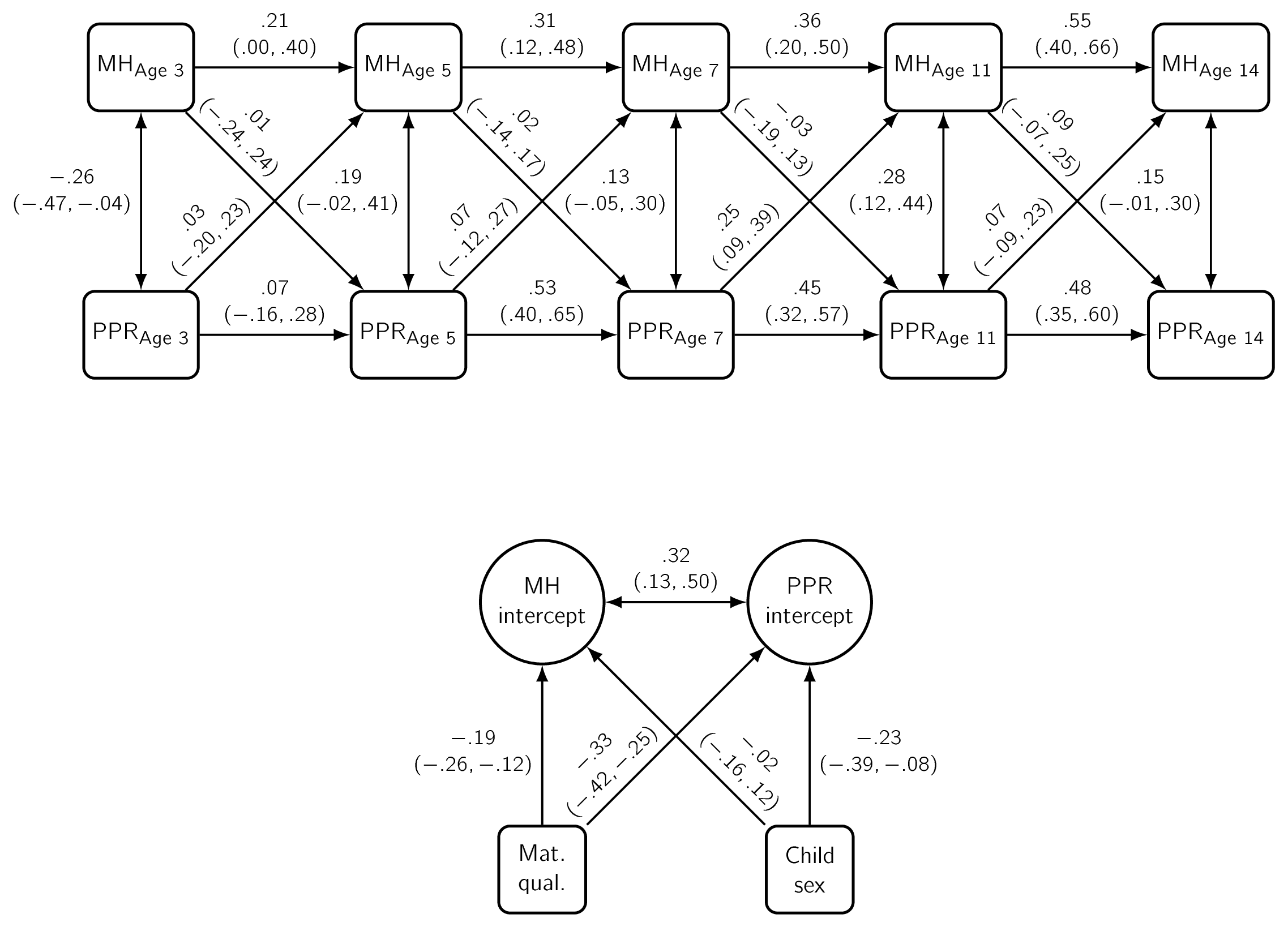


Figure S2: Probit estimates with 95% credible intervals from a random intercept cross-lagged panel model of Peer Problems (PPR) and maternal mental health (MH). *Adjusted for maternal qualifications and child sex. (N=1838)
